# The opposing effect of acute and chronic *Toxoplasma gondii* infection on tumor development

**DOI:** 10.1186/s13071-024-06240-6

**Published:** 2024-06-04

**Authors:** Yining Song, Hao Yuan, Xiaoying Yang, Zipeng Yang, Zhaowen Ren, Shuting Qi, Houjing He, Xiu-Xiang Zhang, Tiantian Jiang, Zi-Guo Yuan

**Affiliations:** 1 College of Veterinary Medicine, South China Agricultural University, Guangzhou, 510642 Guangdong People’s Republic of China; 2 Key Laboratory of Zoonosis Prevention and Control of Guangdong Province, Guangzhou, 510642 Guangdong People’s Republic of China; 3 Key Laboratory of Zoonosis of Ministry of Agriculture and Rural Affairs, South China Agricultural University, Guangzhou, 510642 Guangdong People’s Republic of China; 4 College of Agriculture, South China Agricultural University, Guangzhou, 510642 Guangdong People’s Republic of China; 5 Department of Pediatrics, School of Medicine, University of California, La Jolla, San Diego, CA USA

**Keywords:** *Toxoplasma gondii*, Lewis lung carcinoma, Acute and chronic toxoplasmosis, Mice, Tumor

## Abstract

**Background:**

The interplay between *Toxoplasma gondii* infection and tumor development is intriguing and not yet fully understood. Some studies showed that *T. gondii* reversed tumor immune suppression, while some reported the opposite, stating that *T. gondii* infection promoted tumor growth.

**Methods:**

We created three mouse models to investigate the interplay between *T. gondii* and tumor. Model I aimed to study the effect of tumor growth on *T. gondii* infection by measuring cyst number and size. Models II and III were used to investigate the effect of different stages of *T. gondii* infection on tumor development via flow cytometry and bioluminescent imaging. Mouse strains (Kunming, BALB/c, and C57BL/6J) with varying susceptibilities to tumors were used in the study.

**Results:**

The size and number of brain cysts in the tumor-infected group were significantly higher, indicating that tumor presence promotes *T. gondii* growth in the brain. Acute *T. gondii* infection, before or after tumor cell introduction, decreased tumor growth manifested by reduced bioluminescent signal and tumor size and weight. In the tumor microenvironment, CD4^+^ and CD8^+^ T cell number, including their subpopulations (cytotoxic CD8^+^ T cells and Th1 cells) had a time-dependent increase in the group with acute *T. gondii* infection compared with the group without infection. However, in the peripheral blood, the increase of T cells, including cytotoxic CD8^+^ T cells and Th1 cells, persisted 25 days after Lewis lung carcinoma (LLC) cell injection in the group with acute *T. gondii*. Chronic *T. gondii* infection enhanced tumor growth as reflected by increase in tumor size and weight. The LLC group with chronic *T. gondii* infection exhibited decreased percentages of cytotoxic CD8^+^ T cells and Th1 cells 25 days post-LLC injection as compared with the LLC group without *T. gondii* infection. At week 4 post-LLC injection, chronic *T. gondii* infection increased tumor formation rate [odds ratio (OR) 1.71] in both KM and BALB/c mice.

**Conclusions:**

Our research elucidates the dynamics between *T. gondii* infection and tumorigenesis. Tumor-induced immune suppression promoted *T. gondii* replication in the brain. Acute and chronic *T. gondii* infection had opposing effects on tumor development.

**Graphical Abstract:**

**Figure:**
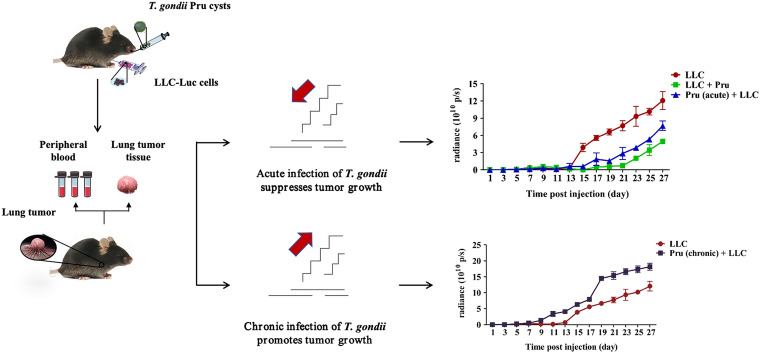

**Supplementary Information:**

The online version contains supplementary material available at 10.1186/s13071-024-06240-6.

## Background

*Toxoplasma gondii* is an important zoonotic protozoan that infects all warm-blooded vertebrates. About 30% of the human population is chronically infected with *T. gondii* [1]. *T. gondii* is fatal in people with compromised immune systems, such as patients with cancer undergoing chemotherapy, patients with acquired immunodeficiency syndrome (AIDS), and organ transplant recipients [2]. Studies have shown that BALB/c and C57BL/6 mice respond to type II *T. gondii* infection differently. The levels of Tim-3/galectin-9, PD-1/PD-L1, and indoleamine-2,3-dioxygenase in these two different mouse breeds showed significant differences post-*T. gondii* infection [3, 4]. These differences might contribute to the resistance of BALB/c and the susceptibility of C57BL/6 to *T. gondii* infection [3, 4]. The current study chose different mouse strains with varying susceptibilities to *T. gondii* to study the interplay between *T. gondii* infection and tumor development.

Cancer is the second leading cause of death in developing countries after cardiovascular disease [5]. It is predicted that the number of cancer deaths worldwide will continue to rise, reaching 11 million by 2030 [6]. There were 2.82 million new cancer cases and 1.96 million cancer deaths in China in 2008, accounting for 22.3% and 25.9% of the world total, respectively. By 2030, the estimated number of new cases and cancer deaths in China will reach 4.87 million and 3.6 million, respectively [4].

A myriad of studies has shown that *T. gondii* infection increases the risk of tumor development and promotes tumor growth. *T. gondii* positivity in patients with cancer is significantly higher than in healthy controls [7–10]. Compared with the 19.67% seroprevalence of anti-*T. gondii* antibodies in control subjects (*P* < 0.05), *T. gondii* seroprevalence was the highest in patients with lung cancer (60.94%), followed by patients with cervical cancer (50.00%), patients with brain cancer (42.31%), and patients with endometrial cancer (41.67%) [11]. *T. gondii* infection can increase the risk of brain cancer by 1.8 times, suggesting that *T. gondii* infection is a risk factor [12]. A controlled study of patients with hematological malignancies showed that the positivity of anti-*T. gondii* immunoglobulin G (IgG) antibodies was 36.6% and 8.7% in patients and healthy controls, respectively. This positivity is not associated with gender and age, which highlights the association between *T. gondii* infection and increased rate of hematological malignancies [13].

However, some studies have shown that *T. gondii* infection hinders disease development, including cancer [14–17]. Intratumoral injection of non-replicating *T. gondii* cps strain in mice led to heightened CD8^+^ T-cell-mediated antitumor response, which eliminated the established melanoma [18]. In another study, cps treatment of mice was shown to reverse tumor-associated immunosuppression to immunostimulation [18]. The invasion of tumor-associated CD11c (+) cells by *T. gondii* cps converted these cells to immunostimulatory phenotypes, leading to antigen cross-presentation and CD8^+^ T cell priming [19]. Intraperitoneal injection of cps induced CD8^+^ T-cell-mediated antitumor response, leading to the rejection of ovarian carcinoma [19]. *T. gondii* ME49 strain inhibited tumor cell replication by activating Th1 cytokines including IFN-γ [20]. Mice with Lewis lung cancer that were infected with *T. gondii* ME49 strain had increased survival rate, higher percentage of CD8^+^ T cells, elevated IFN-γ mRNA expression, serum IgG2a titer, and cytotoxic T lymphocyte response. Furthermore, substantial inhibition of angiogenesis was observed in tumor tissues of mice infected with the ME49 strain, which contributed to the inhibition of tumor growth [21, 22].

This research aims to elucidate the intricate interplay between *T. gondii* infection and the development of lung cancer, particularly investigating how different infection stages of *T. gondii* impact tumor growth. We construct various experimental animal models. To gauge the impact of *T. gondii* infection on tumor growth, we focused on the immunological changes in the peripheral blood and the tumor microenvironment, in addition to measuring tumor size and weight. The significance of this study lies not only in elucidating the interplay between *T. gondii* infection and tumor growth, but also in offering new perspectives for the development of infectious-agent-mediated cancer therapeutics.

## Methods

### Animals

All C57BL/6J, BALB/c, and Kunming (KM) mice were purchased from the Guangdong Medical Laboratory Animal Center and were bred under specific pathogen-free conditions at South China Agricultural University. All animal care procedures were conducted in compliance with NIH guidelines (NIH pub. no. 85-23, revised in 1996) and were approved by the Animal Ethics Committee of South China Agricultural University (no. SCAU2023F276).

### Cell lines

Luciferase labeled Lewis lung carcinoma cells (LLC-Luc) were purchased from Hunan Fenghui Biotechnology Co., Ltd. LLC-Luc were cultured in DMEM-F12, 10% FBS, and 100 units/mL penicillin–streptomycin (all products were from GIBCO™). The final concentration of the injection was adjusted to a dose of 2 × 10^6^ LLC-Luc per 0.2 mL.

### Parasites

KM mice were infected with the type II Prugniaud (Pru) strain preserved in our laboratory. The entire brains of KM mice with chronic infection of *T. gondii* were collected and homogenized in 1 mL saline solution to obtain cysts. A total of 10 μL of brain homogenate were loaded to the hemocytometer for microscopic examination of cyst size and number. This was repeated five times to create five technical replicates. Five biological replicates were generated through sampling the four other mice in the same experimental group. The average cyst size and number were obtained. We used four cysts with an average size of around 40 μm. The number of cysts was adjusted on the basis of the average size of the cyst. If the cyst size was between 50 μm and 80 μm, three cysts were used. Five cysts were used if the size was smaller than 30 μm. This is to ensure that mice are infected but will not die from the infection due to high number of parasites.

We used the same dosage for both acute and chronic infection (model II). The timing of tumor injection was different, however. With the acute model, tumor injection was performed at 5 days post-*T. gondii* infection, and for the chronic model, tumor injection was at 20 days post-*T. gondii* infection.

### Experimental animal model I

**Fig. 1:**
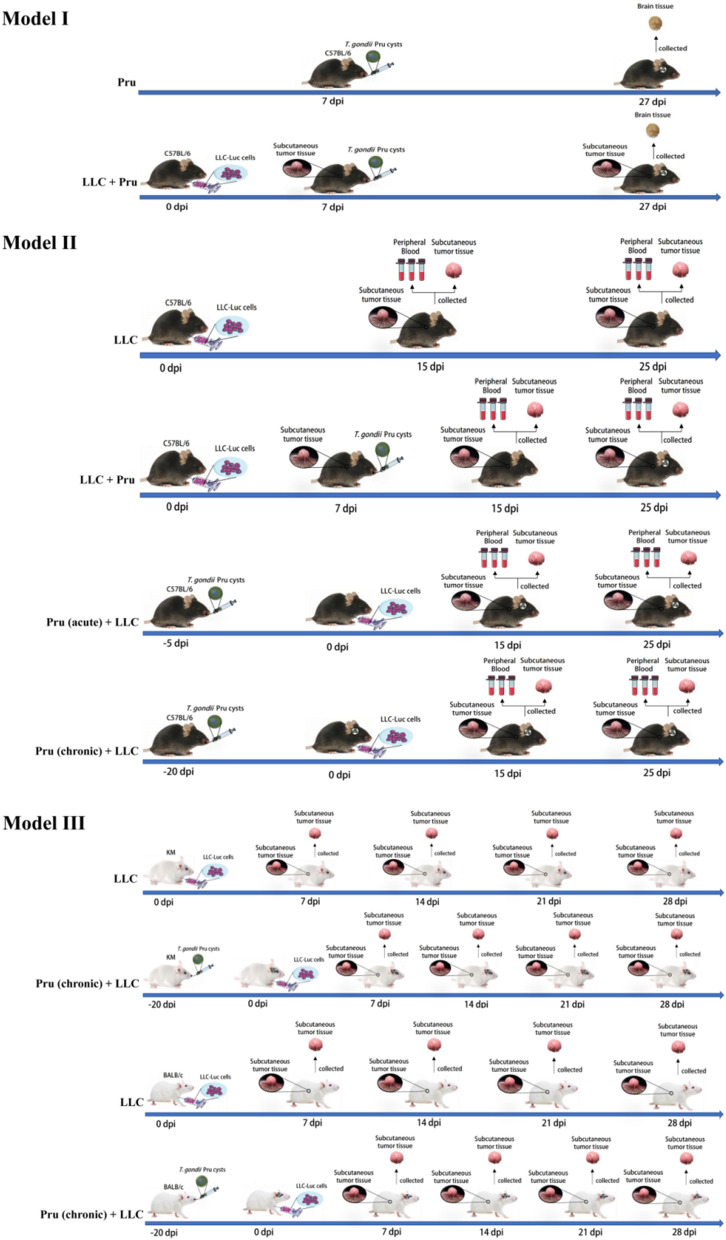
Schematic of the experimental setup for models I, II, and III. Model I was designed to investigate the effect of tumor growth on *T. gondii* infection. For model I, the size and number of cysts were measured. Model II was used for bioluminescence imaging and flow-cytometry analysis to investigate the effect of different stages of *T. gondii* infection on tumor growth. Model III was designed to study the effect of chronic *T. gondii* infection on tumor development in different mouse strains, namely KM mice and BALB/c mice. The day of LLC injection was set as day 0 post-injection (0 dpi)

Three models were created in this research (Fig. 1). For all models, LLC-Luc cells described in the Cell lines section were injected subcutaneously into the axilla of the mice. *T. gondii* cysts described in the Parasites section were administered via oral gavage. With this inoculation method, tumor generally develops around the axilla. Although tumor-bearing animals normally do not die, extreme weakness or infection may occur due to tumor development and skin rupture. In compliance with animal welfare and to ensure the accuracy of the data, the humanitarian endpoint was set at 25 days post-LLC-Luc injection for C57BL/6 mice and 28 days post-LLC-Luc injection for KM mice and BALB/c mice.

Model I was designed for the examination of brain cysts and to investigate the effect of tumor growth on *T. gondii* infection (Fig. 1). In total, ten SPF C57BL/6 mice (females, 35 days old), were randomly divided into two groups of five: (1) Pru group: mice were orally infected with four *T. gondii* cysts, and (2) LLC + Pru group: mice were injected with 2 × 10^6^ LLC-Luc cells followed by oral infection with four *T. gondii* cysts 7 days later.

Then, 20 days post-*T. gondii* infection, the infected C57BL/6 mice were euthanized. Brain tissue was collected and homogenized in 1 mL saline, and 10 µL of brain homogenate were taken for microscopic examination. Five samples were taken from each brain for the enumeration of size and number of brain cysts. This was repeated for each of the five mice in each of the two experimental groups.

### Experimental animal model II

Model II was used for bioluminescence imaging and flow-cytometry analysis. A total of 24 SPF C57BL/6 mice (females, 35 days old) were randomly divided into four groups of six: (1) LLC group: mice were subcutaneously injected with 2 × 10^6^ LLC-Luc cells into the axilla, (2) LLC + Pru group: mice were subcutaneously injected with 2 × 10^6^ LLC-Luc cells into the axilla and orally infected with four *T. gondii* cysts 7 days later, (3) Pru (acute) + LLC group: mice were orally infected with four *T. gondii* cysts and subsequently injected with 2 × 10^6^ LLC-Luc cells into the axilla 5 days post-infection, and (4) Pru (chronic) + LLC group: mice were infected with four *T. gondii* cysts and were subsequently injected with 2 × 10^6^ LLC-Luc cells 20 days later.

### Bioluminescent imaging

Tumor development was monitored using bioluminescence imaging (Fig. 1). Mice injected with LLC-Luc cells were imaged every other day until 27 days post-injection. Each mouse received an intraperitoneal injection of d-luciferin (200 mg/kg) in phosphate buffer saline (PBS). After 15 min, mice were anesthetized via inhalation of isoflurane and imaged using an IVIS Lumina LT system (PerkinElmer Imaging Systems, USA). Data were processed and reconstructed on the workstation.

For C57BL/6J mice, significant differences in bioluminescence among treatment groups were observed at 15 days post-injection (Additional file 1: Fig. S1), with the deaths of animals occurring at 27 days post-injection. In compliance with animal welfare and to ensure the accuracy of the data, we set the humanitarian endpoint at 25 days post-injection. Therefore, C57BL/6J mice were euthanized and sampled at 15 days and 25 days post-injection.

### Flow cytometry

Different classes of CD3^+^ T cells in the subcutaneous tumor tissue were analyzed. Mice in different groups were anesthetized at day 15 and day 25 post-injection of LLC-Luc cells. The orbital venous plexus was punctured, and 100 μL of peripheral blood was collected from each mouse. Mice were then euthanized and dissected. Tumor tissues were collected and immediately rinsed with PBS to rid of blood. Tissue was minced to pieces of around 3–4 mm^2^ and immediately transferred to RPMI 1640 culture medium supplemented with type II DNase (Cat# D4693-1G, Sigma-Aldrich). The mixture was incubated for 2 h at 37 °C with shaking at 65 rpm. The digested tissues were filtered to acquire a suspension of single cells. After the centrifugation at 500×*g* for 5 min, the supernatant was discarded. The pelleted intact cells were collected and resuspended in complete RPMI 1640 media. The concentration of cells were counted on a hemacytometer.

The whole blood collected from each mouse was treated with RBC Lysis Buffer (Cat# 00-4300-54, eBioscience) according to the manufacturer’s instructions. After centrifugation, the pellet was collected and resuspended in RPMI 1640 complete media. The number of white blood cells were counted on a hemacytometer.

Resuspended cells were stained with diluted (1:1000 in PBS) live/dead fixable viability dye (FVD-eFluor506, Cat# 65-0866-14, eBioscience) for 30 min at 4 °C in the dark to differentiate live from dead cells. To block non-specific binding, mouse and human cells were incubated with FcR blocking reagent for 20 min at 4 °C (Cat# 16-0161-86, eBioscience). For intracellular cytokine staining, cells were re-stimulated with a cell stimulation cocktail containing PMA and ionomycin (Cat# 00-4970-93, eBioscience) in the presence of a protein transport inhibitor cocktail containing Brefeldin A and Monensin (Cat# 00-4980-03, eBioscience). Intracellular cytokine staining was performed using the intracellular fixation and permeabilization kit (Cat# 00-5523-00, eBioscience) for 60 min at 4 °C in the dark. Cells were stained with the following antibodies for cell markers (for 30 min at 4 °C in the dark): anti-CD3ε (Cat# 152316, Biolegend), anti-CD8a (Cat# 12-0081-82, eBioscience), anti-CD4 (Cat# 553046, BD), Granzyme B (Cat# 17-8898-82, eBioscience), T-bet (Cat# 25-5825-82, eBioscience), and mouse/rat/human FOXP3 (Cat# 320008, 320014, 320012, Biolegend).

### Experimental animal model III

Model III was designed to observe the tumor growth in different mouse strains, namely KM mice and BALB/c mice. A total of 32 SPF KM mice or BALB/c mice (females, 35 days old) were randomly divided into two groups of 16: (1) LLC group: mice were injected with 2 × 10^6^ LLC-Luc, and (2) Pru (chronic) + LLC group: mice were infected with four *T. gondii* cysts and followed by infection with 2 × 10^6^ LLC-Luc 20 days later.

After injection of LLC-Luc, the tumor growth in each group of mice in animal model III (Fig. 1) were observed weekly. The mice were euthanized 28 days post-LLC infection. The tumor formation rate was determined, and the odds ratio (OR) of tumor formation rate was calculated.

The odds ratio is calculated as OR = $\frac{a/b}{c/d}$, where *a* represents the number of mice with successful tumor engraftment and *T. gondii* infection, and *b* represents the number of mice without successful tumor engraftment but with *T. gondii* infection. The number of mice with successful tumor engraftment but without *T. gondii* infection is represented as *c*, and the number of mice without successful tumor engraftment and without *T. gondii* infection is represented as *d*.

### Statistical analysis

The data were analyzed using a two-tailed unpaired Student’s *t*-test to compare the differences between two independent groups. All data were expressed as means ± standard deviation (SD). The significance level was set at *P < 0.05, **P < 0.01, ***P < 0.001 and ****P < 0.0001.

## Results

### Experimental design

Due to the complex nature of the experimental design of this study, we constructed a diagram to facilitate the understanding of the experimental setup (Fig. 1). Namely, three models were designed, and each was intended to address a different question. Model I was designed to assess the impact of LLC infection on *T. gondii* growth by measuring the number and size of brain cysts. In model I, tumor growth was allowed to proceed the *T. gondii* infection. Model II was designed to investigate the effects of different stages of *T. gondii* infection on tumor growth via bioluminescence imaging and flow cytometry. In addition, 15 days and 25 days post-LLC injection were chosen as sampling dates because significant bioluminescence was detected at these timepoints (Additional file 1: Fig. S1). The abundance of Th1 cells and cytotoxic CD8^+^ T cells in the tumor microenvironment (TME) and peripheral blood were analyzed 15 days and 25 days post-LLC cell injection via flow cytometry. Model III aimed to address the effect of chronic *T. gondii* infection on tumor formation in KM mice and BALB/c mice which had varying tumor susceptibility.

### Tumor growth exacerbates *T. gondii* infection (model I)

**Fig. 2:**
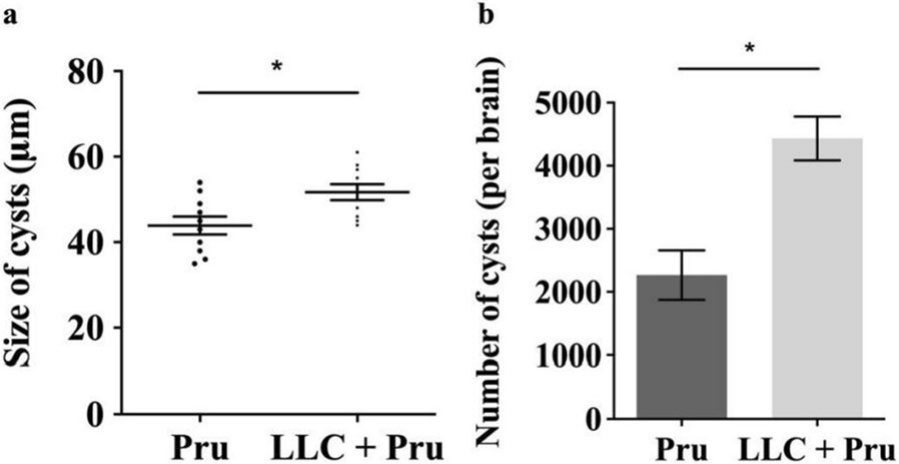
Tumor growth promotes the replication of *T. gondii* (model I). **a** Average size in diameter of brain cysts from mice in Pru and LLC + Pru groups 20 days post-*T. gondii* infection. **b** Number of cysts per brain in Pru and LLC + Pru groups 20 days post-*T. gondii* infection. Data are shown as mean ± SD (*P < 0.05)

To understand the effect of tumor on the infection of *T. gondii*, the number and size of brain cysts in the Pru and the LLC + Pru groups were measured 20 days post-*T. gondii* infection (model I). We found that the number and size of brain cysts in the LLC + Pru group were significantly bigger than those in the Pru group (Fig. 2a, b), which indicated that the development of tumor promoted the growth of *T. gondii* in host central nervous system (CNS).

### Acute infection of *T. gondii* suppresses tumor growth (model II)

**Fig. 3:**
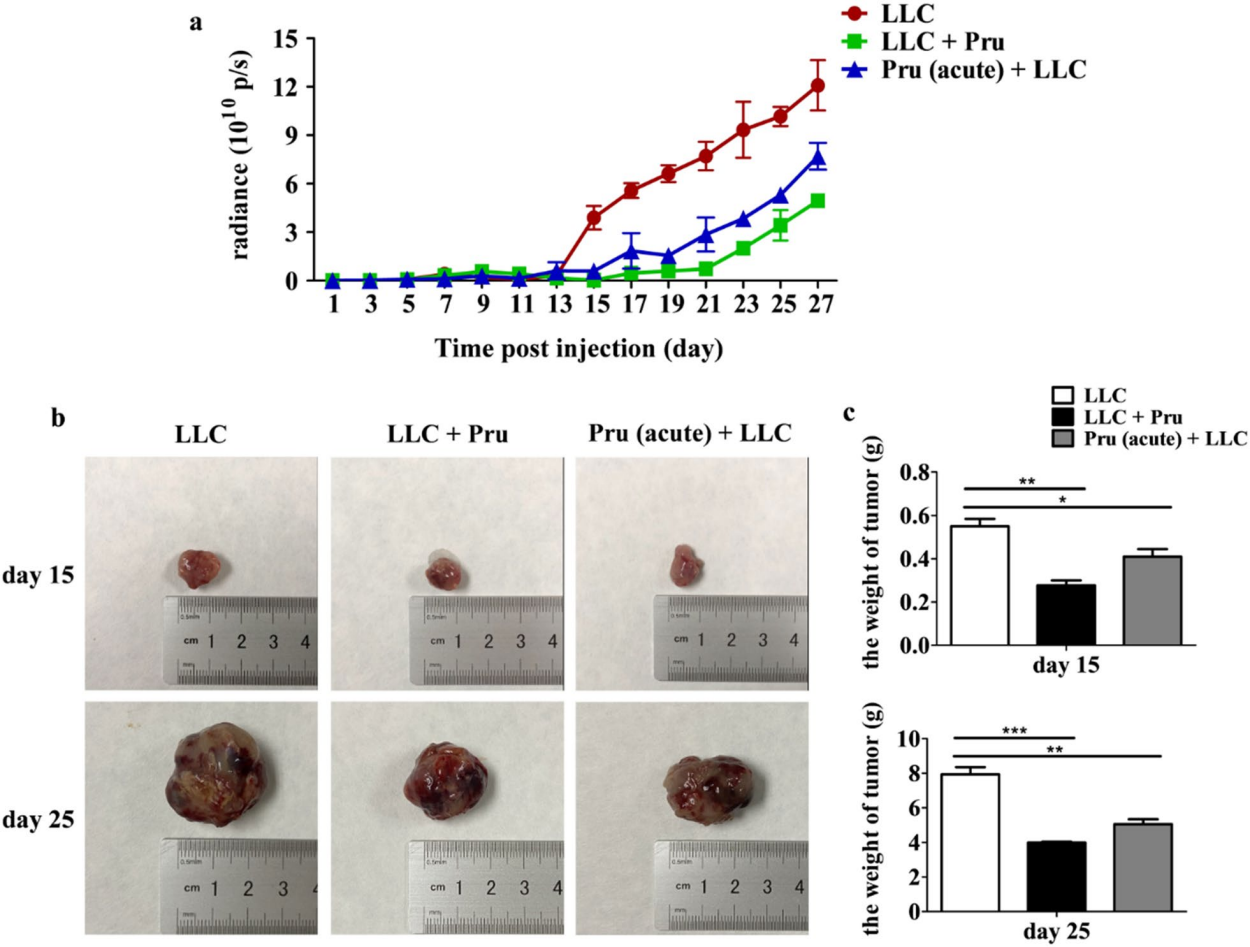
Acute infection of *T.  gondii* inhibits tumor growth (model II). **a** Luminescence intensity from bioluminescence imaging of live mice injected with LLC-Luc cells. **b** Tumor photos were taken from mice 15 days and 25 days post-injection of LLC-Luc cells. **c** Tumor weight was measured 15 days and 25 days post-injection of LLC-Luc cells. Data are shown as mean ± SD (*P < 0.05, **P < 0.01, ***P < 0.001)

The mice were observed from day 1 to day 27 after LLC-luc cell injection (Fig. 3a). At 15 days and 25 days post-LLC-Luc injection, the tumors were collected, photographed, and weighed. The weight of the tumor in the LLC + Pru or the Pru (acute) + LLC group was significantly smaller than that in the LLC groups (Fig. 3). These data indicate that acute *T. gondii* infection hinders tumor growth regardless of the order of infection.

### Acute *T. gondii* infection induced time-dependent reversal of the immunosuppression in TME (model II)

**Fig. 4:**
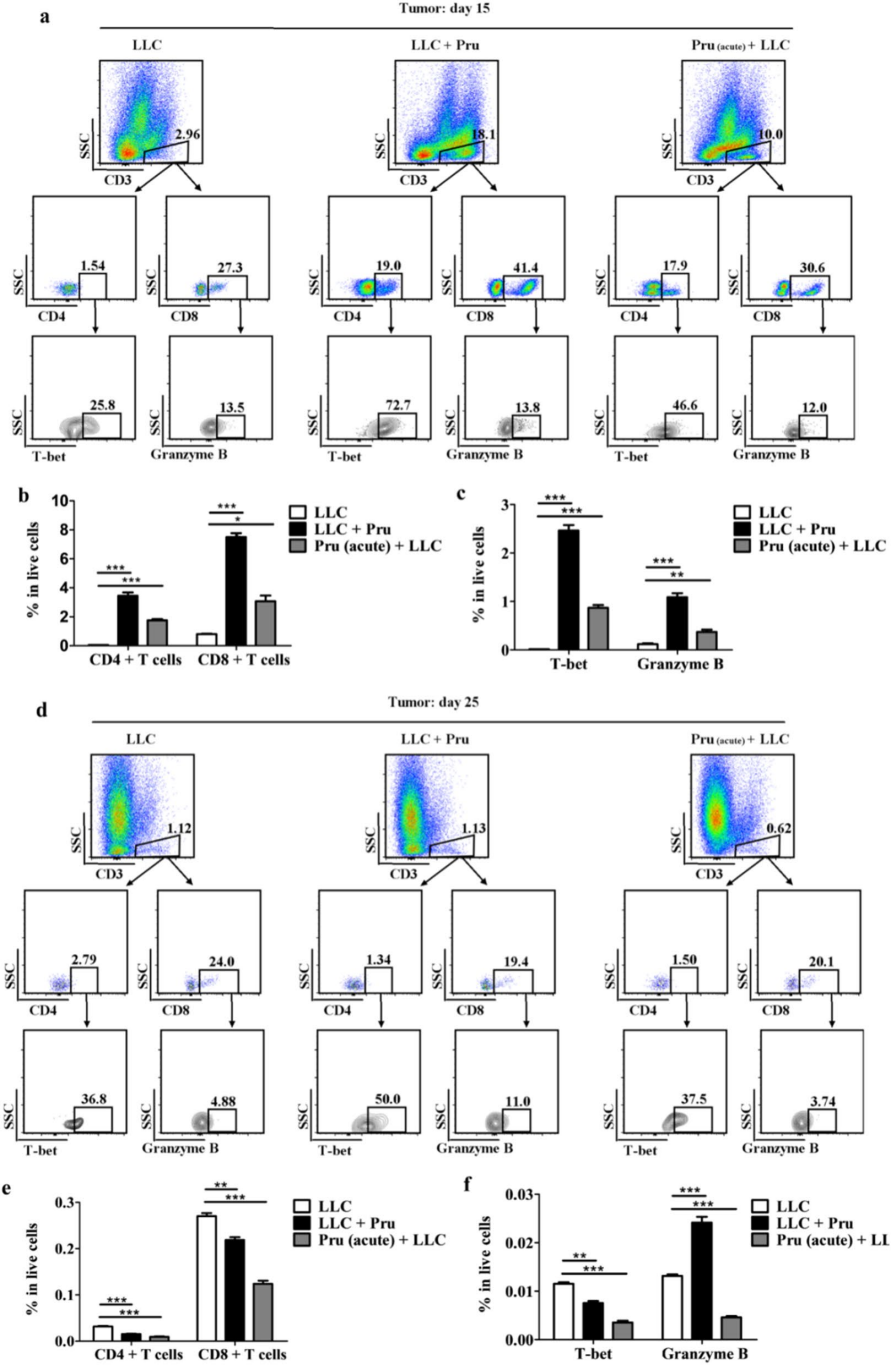
Acute *T. gondii* infection induces time-dependent reversal of the immunosuppression in TME (model II). **a**, **d** Gating scheme for the selection of Th1 cells and cytotoxic CD8^+^ T cells from the tumor samples collected 15 days and 25 days post-LLC-Luc injection. **b**, **e** Percentages of CD4^+^ and CD8^+^ T cells 15 days and 25 days post-injection. **c**, **f** Percentages of Th1 cells and cytotoxic CD8^+^ T cells 15 days and 25 days post-injection. Data are shown as mean ± SD (*P < 0.05, **P < 0.01, ***P < 0.001)

To understand the immune response in different infection groups, we analyzed the abundance of Th1 cells (T-bet positive CD4^+^) and cytotoxic CD8^+^ T cells (granzyme B^+^) in tumor (Fig. 4) and peripheral blood (Additional file 1: Fig. S2) of mice in the LLC, LLC + Pru, and Pru (acute) + LLC groups via flow cytometry 15 days and 25 days post-LLC injection (model II). Then, 15 days post-LLC injection, the percentages of effector T cells including Th1 and cytotoxic CD8^+^ T cells were significantly higher in LLC + Pru and Pru (acute) + LLC groups than those in LLC group, with LLC + Pru group having higher proportions of effector T cells than Pru (acute) + LLC group (Fig. 4b, c). However, this increase of effector T cells failed to persist over time, and 25 days post-LLC injection, although the percentages of all cell populations plummeted (Fig. 4e, f), the percentage of cytotoxic CD8^+^ T cells in the LLC + Pru group was still higher than that in LLC group or Pru (acute) + LLC group (Fig. 4f). In the peripheral blood, however, the proportions of effector T cells including their subpopulations (Th1 cells and cytotoxic CD8^+^ T cells) in the LLC + Pru and Pru (acute) + LLC were consistently higher 15 days and 25 days post-tumor cell injection (Additional file 1: Fig. S2).

### Chronic infection of *T. gondii* promotes tumor growth (model II)

**Fig. 5:**
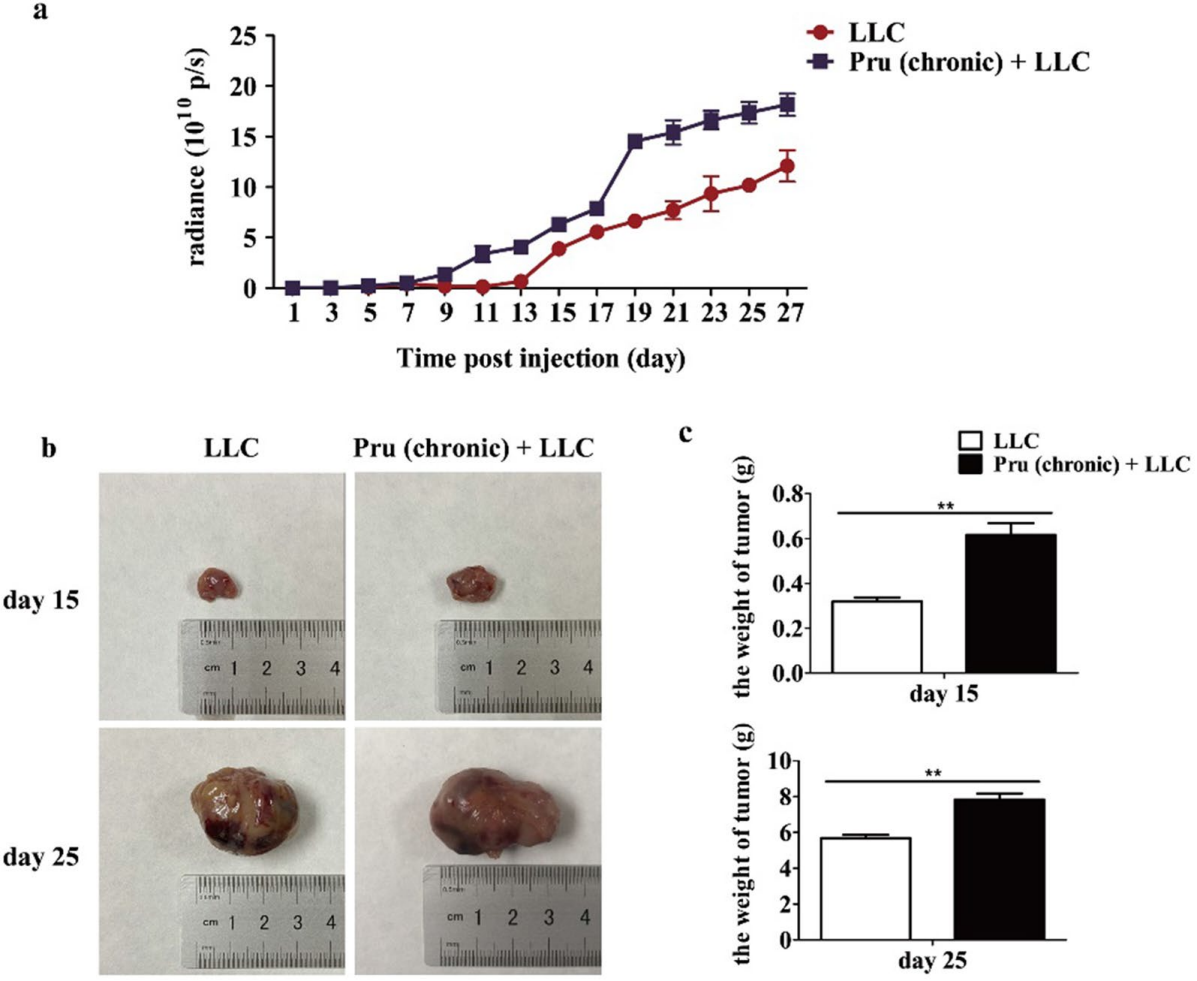
Chronic infection of *T. gondii* promotes tumor growth (model II). **a** Bioluminescence imaging to monitor tumor cell growth. Data were recorded every other day from day 1 to day 27 post-LLC injection. **b** Tumor photos were taken 15 days and 25 days post-injection of LLC. **c** Tumor weight was measured 15 days and 25 days following LLC inoculation. Data are represented as means ± SD (**P < 0.01)

To understand the effect of chronic *T. gondii* infection on tumor growth, the tumor growth in LLC and Pru (chronic) + LLC groups was monitored from day 1 to day 27 post-LLC inoculation (model II). A significantly higher luciferase signal was found in the group with chronic *T. gondii* infection (Additional file 1: Fig. S1a). Tumors from each group were photographed and weighed 15 days and 25 days post-LLC inoculation. The data showed that tumor from Pru (chronic) + LLC group was significantly larger than that in the LLC group, which indicates that chronic infection of *T. gondii* promoted tumor growth (Fig. 5b, c).

### Chronic infection of *T. gondii* enhanced TME immunosuppression (model II)

**Fig. 6:**
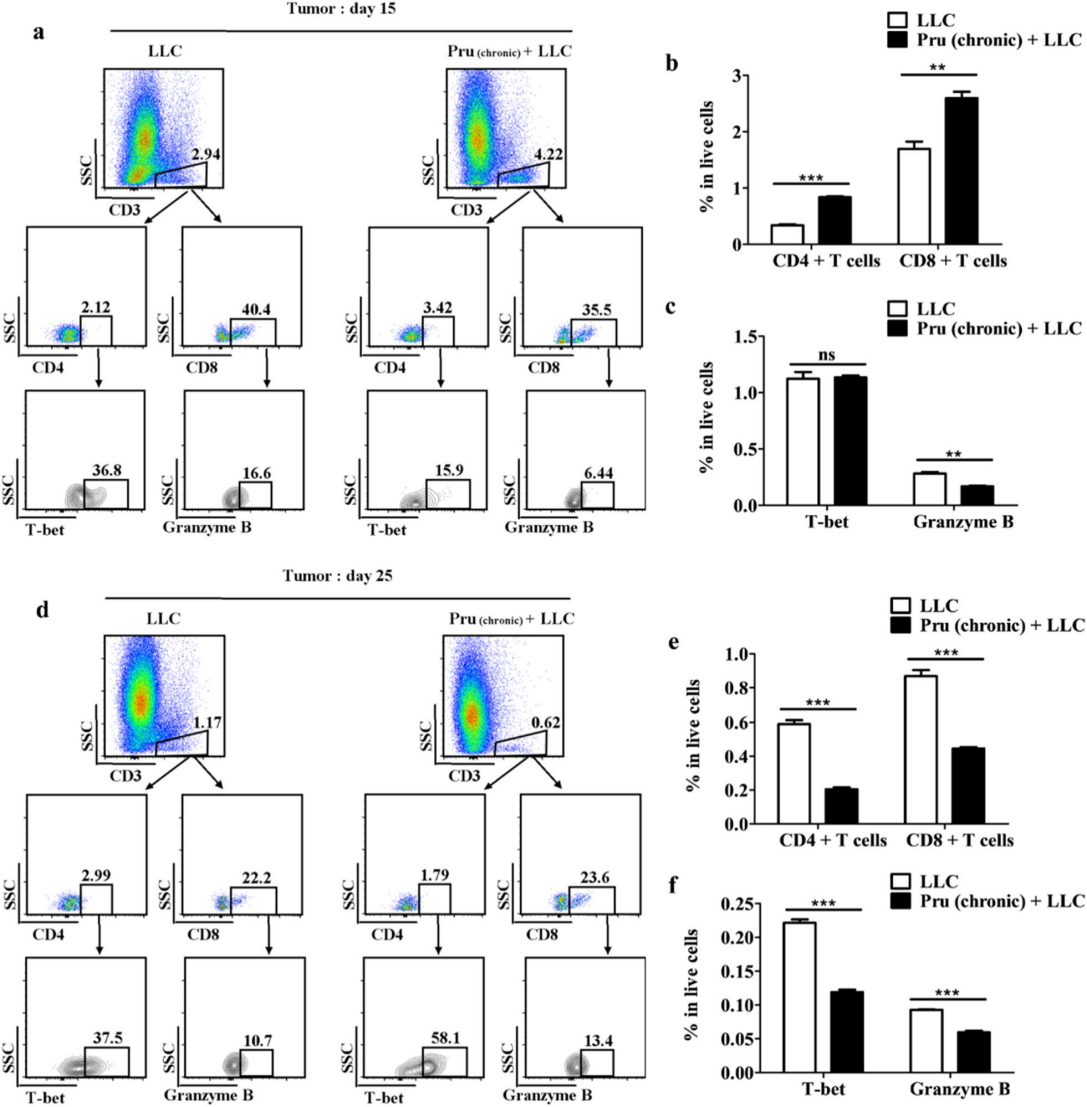
Chronic infection of *T. gondii*-enhanced TME immunosuppression (model II). **a**, **d** Gating schemes for the selection of CD4^+^ and CD8^+^ T cells as well as Th1 cells and cytotoxic CD8^+^ T cells from tumor samples. **b**, **e** Percentages of CD4^+^ and CD8^+^ T cells in the tumor microenvironment. **c**, **f** Percentages of Th1 cells and cytotoxic CD8^+^ T cells in the tumor microenvironment. Data are denoted as mean ± SD (**P < 0.01, ***P < 0.001)

To understand how chronic infection of *T. gondii* affects tumor growth, we analyzed the numbers of effector T cells in tumor (Fig. 6) and peripheral blood (Additional file 1: Fig. S3) of mice in LLC and Pru (chronic) + LLC groups 15 days and 25 days following injection of LLC cells (model II). By day 15, the Pru (chronic) + LLC group had lower percentage of cytotoxic CD8^+^ T cells (Fig. 6b, c), although no significant difference was observed in the number of Th1 cells. Similarly, 25 days post-infection, the percentages of CD4^+^ and CD8^+^ T cells and their subpopulations (Th1 and cytotoxic CD8^+^ T cells) in the Pru (chronic) + LLC group were significantly lower than those in the LLC group (Fig. 6e, f). However, in the peripheral blood, the numbers of effector T cells in Pru (chronic) + LLC group were higher compared with those in the LLC group (Additional file 1: Fig. S3).

### Chronic infection of *T. gondii* increases tumor formation rate (model III)

**Fig. 7:**
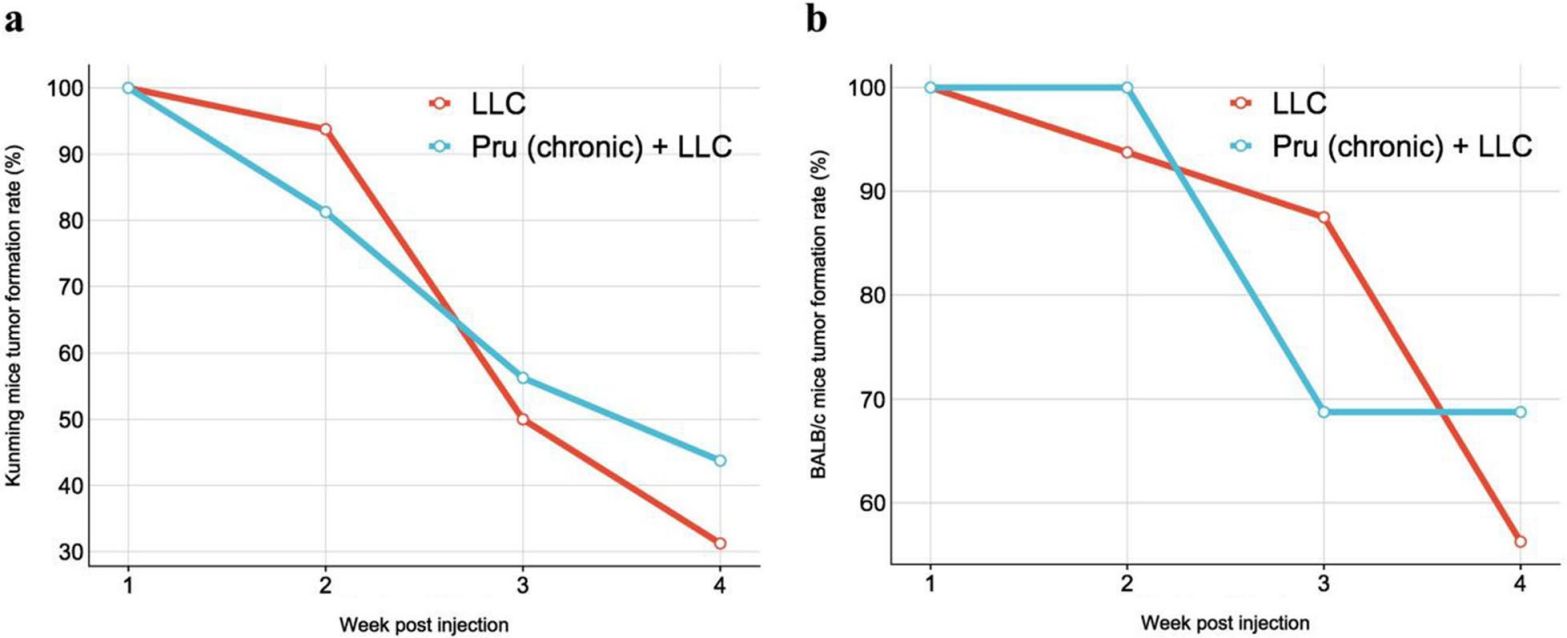
The rate of tumor development in the infected mice (model III). **a** Tumor formation rate in KM mice from week 1 to week 4 after LLC-Luc inoculation. **b** Tumor formation rate in BALB/c mice from week 1 to week 4 after injection of LLC-Luc

KM mice and BALB/c mice with chronic infection of *T. gondii* were injected with LLC-Luc. The tumor formation was monitored weekly, and the mice were euthanized 4 weeks later (model III). For KM mice, the weekly tumor rates were 100%, 93.75%, 50.00%, and 31.25% in the LLC group, and 100%, 81.25%, 56.25%, and 43.75% in the Pru (chronic) + LLC group (Additional file 1: Table S2, Fig. 7a). For BALB/c mice, the weekly tumor rates in the LLC group were 100%, 93.75%, 87.5%, and 56.25%, and 100%, 100%, 68.75%, and 68.75% in the Pru (chronic) + LLC group (Additional file 1: Table S2, Fig. 7b). In both breeds of mice, tumor rate in Pru (chronic) + LLC group was higher than that in LLC group at week 4. In addition, the OR of tumor rate at week 4 was 1.71 in both KM and BALB/c mouse groups, which indicates that the chronic infection of *T. gondii* increases tumor formation rate (Table 1).

**Table 1:** Tumor formation rates in KM mice and BALB/c mice (model III)

Chronic *T. gondii* infection	KM tumor formation				BALB/c tumor formation			
	No. tested	No. neoplasia	% (95% CI)	OR	No. tested	No. neoplasia	% (95% CI)	OR
Positive	16	7	43.75 (23.1, 66.82)	1.71	16	11	68.75 (44.4, 85.84)	1.71
Negative	16	5	31.25 (14.16, 55.6)		16	9	56.25 (33.18, 76.9)	

Tumorigenicity statistics and OR analysis of KM mice and BALB/c mice at week 4 post-LLC injection with chronic *T. gondii* infection

## Discussion

*Toxoplasma gondii* was shown to have an inhibitory effect on various tumors (melanoma, ovarian, lung, or pancreatic tumor) and can improve the survival rate and prolong the survival time of tumor-bearing mice [23, 24]. However, a pro-tumor effect of *T. gondii* infection was also reported [25, 26]. In this study we observed increased number and size of brain cysts in the LLC + Pru group as compared with the Pru group. This indicates that tumor development promotes tachyzoite invasion of CNS and its replication. The replication of tachyzoite is a highly synchronized process. With time, as the number of tachyzoites increases, the size of parasitophorous vacuole proportionally increases [27–29]. Unlike tachyzoite, the proliferation of bradyzoite is asynchronous. The size of the parasitophorous vacuole does not always proportionally increase with the number of bradyzoites, although the proportion of larger cysts increases and that of smaller ones decreases [30]. This allows us to use the size of cysts to gauge the timing of cyst formation in the brain. We found that the average size of brain cysts in the LLC + Pru group was significantly bigger than that in the Pru group, suggesting an earlier entry of the tachyzoites into the CNS in the LLC + Pru group in addition to the possibility of enhanced growth under tumor induced immune suppression.

Inflammation plays a vital role in tumorigenesis. Prolonged inflammation can provide an environment that promotes tumor cell proliferation, survival, angiogenesis, metastasis, and tissue remodeling [31]. However, acute inflammation often triggers an antitumor response, including enhanced antigen presentation and dendritic cell maturation, and leads to tumor cell destruction [32]. While Th2-mediated chronic inflammation promotes tumor growth, Th1-driven acute inflammation contributes to tumor suppression [33]. *T. gondii* strongly induces Th1-driven immune response with the upregulation of IL-12 and IFN-γ [34, 35]. Acute *T. gondii* infection activated Th1 driven antitumor immune response, including significantly enhancing the level of IFN-γ, one of its major cytokines [20].

Immune evasion is fully developed by the time of tumor formation. At this stage, tumor cells evade the attack of the immune system by reducing immune cell recognition, inducing immune cell dysfunction and inhibiting immune cell infiltration into tumors [36]. Cytotoxic CD8^+^ T cells are one of the major antitumor immune cells. Most studies associate the infiltration of CD8^+^ T cells in the TME with good prognosis. CD4^+^ T cell infiltration into the TME can also significantly alter the content, phenotype, and function of myeloid cells [37]. Cancer is known to induce abnormal hematopoiesis with hematopoietic stem cells and progenitor cells often found in the peripheral blood of patients [38]. In tumor-bearing patients, there is an aberrant increase of immature neutrophils and monocytes in the peripheral blood, which eventually migrate to tumor microenvironment (TME) and participate in local immunosuppression [38].

To explore the effect of *T. gondii* acute infection on tumorigenesis, tumor formation was assessed via in vivo imaging, tumor diameter, and weight measurement (Fig. 3). Acute *T. gondii* infection developed after tumor cell introduction (LLC + Pru) had a stronger inhibitory effect on tumor formation, as compared with acute *T. gondii* infection before tumor cell introduction [Pru (acute) + LLC]. In our study, we found that acute infection of *T. gondii* caused time-dependent activation of T cell immune response in tumor-bearing mice. In the tumor microenvironment, we found an increase of CD4^+^ T, CD8^+^ T cells, and Th1 and cytotoxic CD8^+^ T cells at day 15 and a drastic decrease at day 25 post-injection of LLC-Luc (Fig. 4). This phenomenon was more pronounced in LLC + Pru group than the Pru (acute) + LLC group. This is likely due to the timing of *T. gondii* infection, as *T. gondii* induced T cell response wanes with time. In the LLC + Pru group, 8 days and 18 days post-*T. gondii* infection, the samples were taken for analysis, while in the Pru (acute) + LLC group, the time gaps between *T. gondii* infection and tissue sampling were 20 days and 30 days. Nonetheless, the percentage of cytotoxic CD8^+^ T cells in the LLC + Pru group was still significantly higher, at 25 days post-injection. In the peripheral blood, the increase in the proportions of CD4^+^ T cells and CD8^+^ T cells including their subpopulations persisted 25 days post-LLC injection (Additional file 1: Fig. S2). Taken together, our study showed that acute *T. gondii* infection induced T cell immune response, which contributed to the suppression of tumor growth.

*Toxoplasma gondii* is known to manipulate the response of NK cells and T cells, as well as the production of cytokines such as IFN-γ and IL-12, which might impact the host’s ability to detect and control the growth of cancer cells [39–44]. Tumor growth was enhanced in *T. gondii* chronic infection group as reflected by the weight and size of the tumor as well as the number of tumor cells measured by bioluminescence. At 15 days post-tumor cell injection, the cytotoxic CD8^+^ T cells in the Pru (chronic) + LLC were lower than those in the LLC group. However, by day 25, the percentage of cytotoxic CD8^+^ T cells plummeted in both groups regardless of *T. gondii* infection, indicating a tumor-induced T cell immunosuppression. Lower percentages of CD4^+^ and CD8^+^ T cells and Th1 and cytotoxic CD8^+^ T cells were observed in Pru (chronic) + LLC (Fig. 6). *T. gondii* chronic infection [Pru (chronic) + LLC] showed a stronger Th1-type immune response in the periphery blood compared with the LLC group (Additional file 1: Fig. S3). Taken together, the immunosuppressive state in the chronic infection group [Pru (chronic) + LLC] was exacerbated, thereby promoting tumor growth (Fig. 5). Odds ratio analysis (Table 1) also showed that *T. gondii* chronic infection is positively corrected with tumor formation and is a risk factor of tumor development. Taken together, chronic *T. gondii* infection enhanced tumor growth and further suppressed effector T-cell-mediated immune response.

Survival rate was not used as a parameter for evaluation in this study. This is because tumor-bearing is extremely painful for mice, especially at later stages. Tumor-bearing could also cause complications such as microbial infection upon skin rupture, which could skew data. To alleviate animal suffering and ensure accuracy of data, we chose to euthanize mice 25 days or 28 days post-tumor cell injection. In this study, cytokine and other immune modulators in tumor microenvironment and peripheral blood were not studied. Future research should investigate the underlying mechanisms that drive the phenotypes seen in the study.

## Conclusions

We constructed various experimental animal models to gauge the impact of *T. gondii* infection on tumor growth, and focused on the immunological changes in peripheral blood and tumor microenvironment in addition to measuring tumor size and weight. We elucidated the dynamics between *T. gondii* infection and tumorigenesis. The immune suppression induced by tumor promoted *T. gondii* replication in the brain. Acute and chronic *T. gondii* infection had opposing effects on tumor development.

### Supplementary Information

**Additional file 1: Figure S1.** Significance analysis of luminescence intensity from bioluminescence imaging of live mice injected with LLC-Luc cells (model III). (a) Data of LLC, LLC + Pru and Pru (acute) + LLC were recorded every other day from day 1 to day 27 post-LLC injection. (b) Data of LLC and Pru (chronic) + LLC were recorded every other day from day 1 to day 27 post-LLC injection. Data are means ± SD (*P < 0.05, **P < 0.01, ***P < 0.001). **Figure S2.** The effect of *T. gondii* acute infection on T cells in peripheral blood (model III). (a) On day 15 after the mice were injected LLC cells, CD3^+^ T cells in peripheral blood accounted for the proportion of total cells; CD4^+^ T cells as a percentage of CD3^+^ T cells; CD8^+^ T cells as a percentage of CD3-T cells; Th1 CD4^+^ T cells as a percentage of CD4^+^ T cells; and Granzyme B as a percentage of CD8^+^ T cells. (b) On day 15 after the mice were injected with LLC cells, CD4^+^ T cells in peripheral blood accounted for the proportion of total cells; CD8^+^ T cells accounted for the proportion of total cells. (c) On day 15 after the mice were injected with LLC cells, Th1^+^ CD4^+^ T cells in peripheral blood accounted for the proportion of total cells; and Granzyme B accounted for the proportion of total cells. (d) On day 25 after the mice were injected with LLC cells, CD3^+^ T cells in peripheral blood accounted for the proportion of total cells; CD4^+^ T cells as a percentage of CD3^+^ T cells; CD8^+^ T cells as a percentage of CD3^−^ T cells; Th1 CD4^+^ T cells as a percentage of CD4^+^ T cells; Granzyme B as a percentage of CD8^+^ T cells. (e) On day 25 after the mice were injected with LLC cells, CD4^+^ T cells in peripheral blood accounted for the proportion of total cells; CD8^+^ T cells accounted for the proportion of total cells. (f) On day 25 after the mice were injected with LLC cells, Th1 CD4^+^ T cells accounted for the proportion of total cells; and Granzyme B accounted for the proportion of total cells. Data are means ± SD (**P < 0.01, ***P < 0.001). **Figure S3.** The percentages of on effector T cells in peripheral blood (model III). (a) On day 15 after the mice were injected LLC cells, CD3^+^ T cells in peripheral blood accounted for the proportion of total cells; CD4^+^ T cells as a percentage of CD3^+^ T cells; CD8^+^ T cells as a percentage of CD3-T cells; Th1 CD4^+^ T cells as a percentage of CD4^+^ T cells; Granzyme B as a percentage of CD8^+^ T cells. (b) On day 15 after the mice were injected with LLC cells, CD4^+^ T cells in peripheral blood accounted for the proportion of total cells; CD8^+^ T cells accounted for the proportion of total cells. (c) On day 15 after the mice were injected with LLC cells, Th1 CD4^+^ T cells in peripheral blood accounted for the proportion of total cells; and Granzyme B accounted for the proportion of total cells. (d) On day 25 after the mice were injected with LLC cells, CD3^+^ T cells in peripheral blood accounted for the proportion of total cells; CD4^+^ T cells as a percentage of CD3^+^ T cells; CD8^+^ T cells as a percentage of CD3^−^ T cells; Th1 CD4^+^ T cells as a percentage of CD4^+^ T cells; Granzyme B as a percentage of CD8^+^ T cells. (e) On day 25 after the mice were injected with LLC cells, CD4^+^ T cells in peripheral blood accounted for the proportion of total cells; CD8^+^ T cells accounted for the proportion of total cells. (f) On day 25 after the mice were injected with LLC cells, Th1 CD4^+^ T cells accounted for the proportion of total cells; and Granzyme B accounted for the proportion of total cells. Data are means ± SD (*P < 0.05, **P < 0.01, ***P < 0.001). **Figure S4.** Tumor images were captured from three C57BL/6J mice 15 days and 25 days post-injection of LLC-Luc cells.(a) Acute infection of *T. gondii* inhibits tumor growth (model II). (b) Chronic infection of *T. gondii* promotes tumor growth (model II). **Figure S5.** Luminescence intensity pictures from bioluminescence imaging of live mice injected with LLC-Luc cells. (a) Bioluminescent imaging of tumor progression in LLC, LLC + Pru, and Pru (acute) + LLC groups. (b) Bioluminescent imaging of tumor progression in LLC and Pru (chronic) + LLC groups. **Table S1.** Quantitative real-time PCR specific primers set (model II). **Table S2.** Four-week tumor formation rates in KM mice and BALB/c mice (model III).

## Data Availability

All data generated or analysed during this study are included in this published article.
