# Supplementary material for: The opposing effect of acute and chronic Toxoplasma gondii infection on tumor development
Source: Parasit Vectors. 2024 Jun 4;17:247. doi: 10.1186/s13071-024-06240-6 (PMC11149184; doi:10.1186/s13071-024-06240-6)
Supplement: Supplementary file 1 — Additional file 1: Figure S1. Significance analysis of luminescence intensity from bioluminescence imaging of live mice injected with LLC-Luc cells (model III). (a) Data of LLC, LLC + Pru and Pru (acute) + LLC were recorded every other day from day 1 to day 27 post-LLC injection. (b) Data of LLC and Pru (chronic) + LLC were recorded every other day from day 1 to day 27 post-LLC injection. Data are means ± SD (*P < 0.05, **P < 0.01, ***P < 0.001). Figure S2. The effect of T. gondii acute infection on T cells in peripheral blood (model III). (a) On day 15 after the mice were injected LLC cells, CD3+ T cells in peripheral blood accounted for the proportion of total cells; CD4+ T cells as a percentage of CD3+ T cells; CD8+ T cells as a percentage of CD3-T cells; Th1 CD4+ T cells as a percentage of CD4+ T cells; and Granzyme B as a percentage of CD8+ T cells. (b) On day 15 after the mice were injected with LLC cells, CD4+ T cells in peripheral blood accounted for the proportion of total cells; CD8+ T cells accounted for the proportion of total cells. (c) On day 15 after the mice were injected with LLC cells, Th1+ CD4+ T cells in peripheral blood accounted for the proportion of total cells; and Granzyme B accounted for the proportion of total cells. (d) On day 25 after the mice were injected with LLC cells, CD3+ T cells in peripheral blood accounted for the proportion of total cells; CD4+ T cells as a percentage of CD3+ T cells; CD8+ T cells as a percentage of CD3− T cells; Th1 CD4+ T cells as a percentage of CD4+ T cells; Granzyme B as a percentage of CD8+ T cells. (e) On day 25 after the mice were injected with LLC cells, CD4+ T cells in peripheral blood accounted for the proportion of total cells; CD8+ T cells accounted for the proportion of total cells. (f) On day 25 after the mice were injected with LLC cells, Th1 CD4+ T cells accounted for the proportion of total cells; and Granzyme B accounted for the proportion of total cells. Data are means ± SD (**P < 0.01, ***P [file 13071_2024_6240_MOESM1_ESM.docx]

Additional file


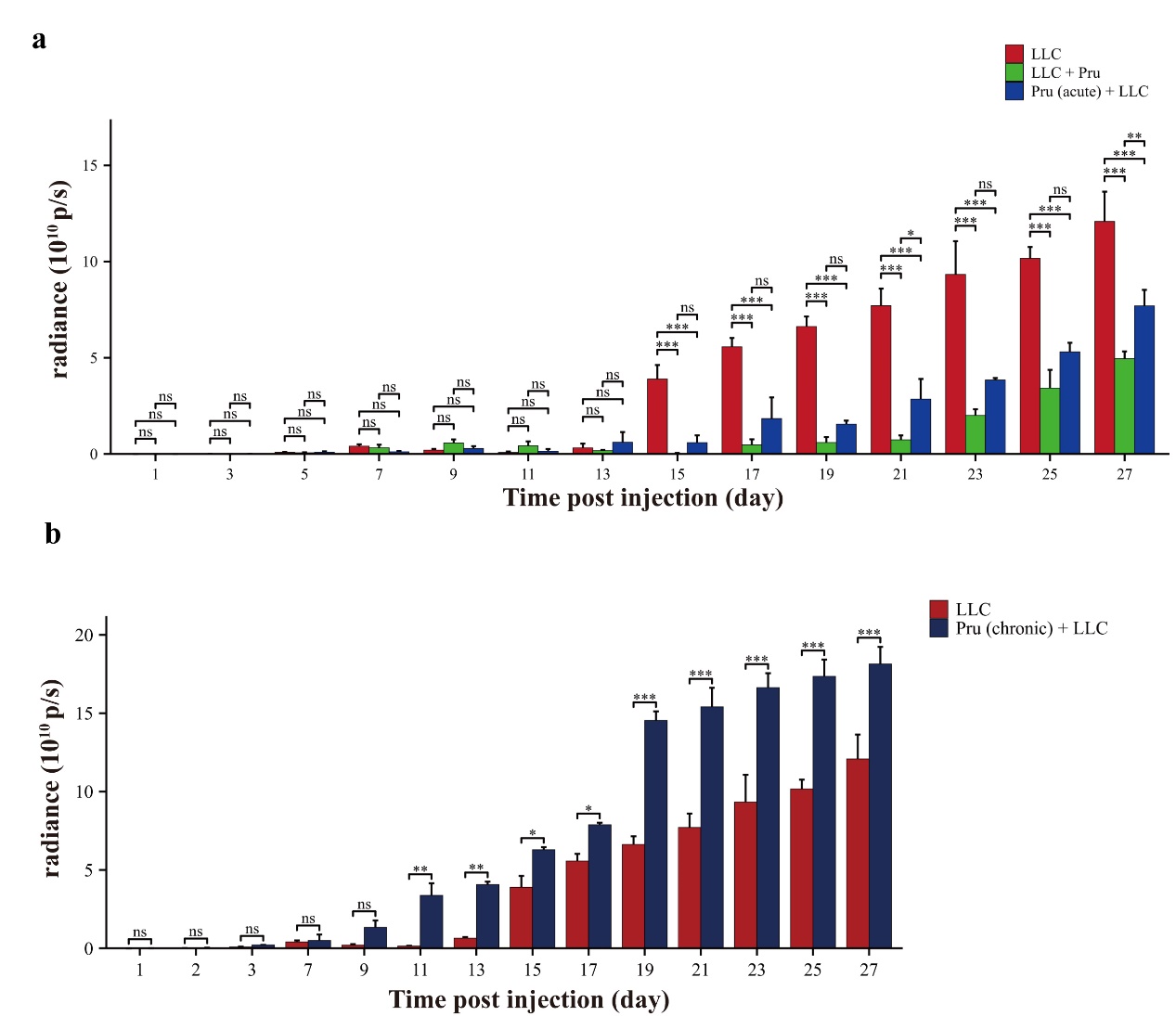


**Fig. S1.** Significance analysis of luminescence intensity from bioluminescence imaging of live mice injected with LLC-Luc cells (model III). (a) Data of LLC, LLC+Pru and Pru (acute) +LLC were recorded every other day from day 1 to day 27 post LLC injection. (b) Data of LLC and Pru (chronic) +LLC were recorded every other day from day 1 to day 27 post LLC injection. Data are means ± SD (^*^ *P* < 0.05, ^**^ *P* < 0.01, ^***^ *P* < 0.001).

**
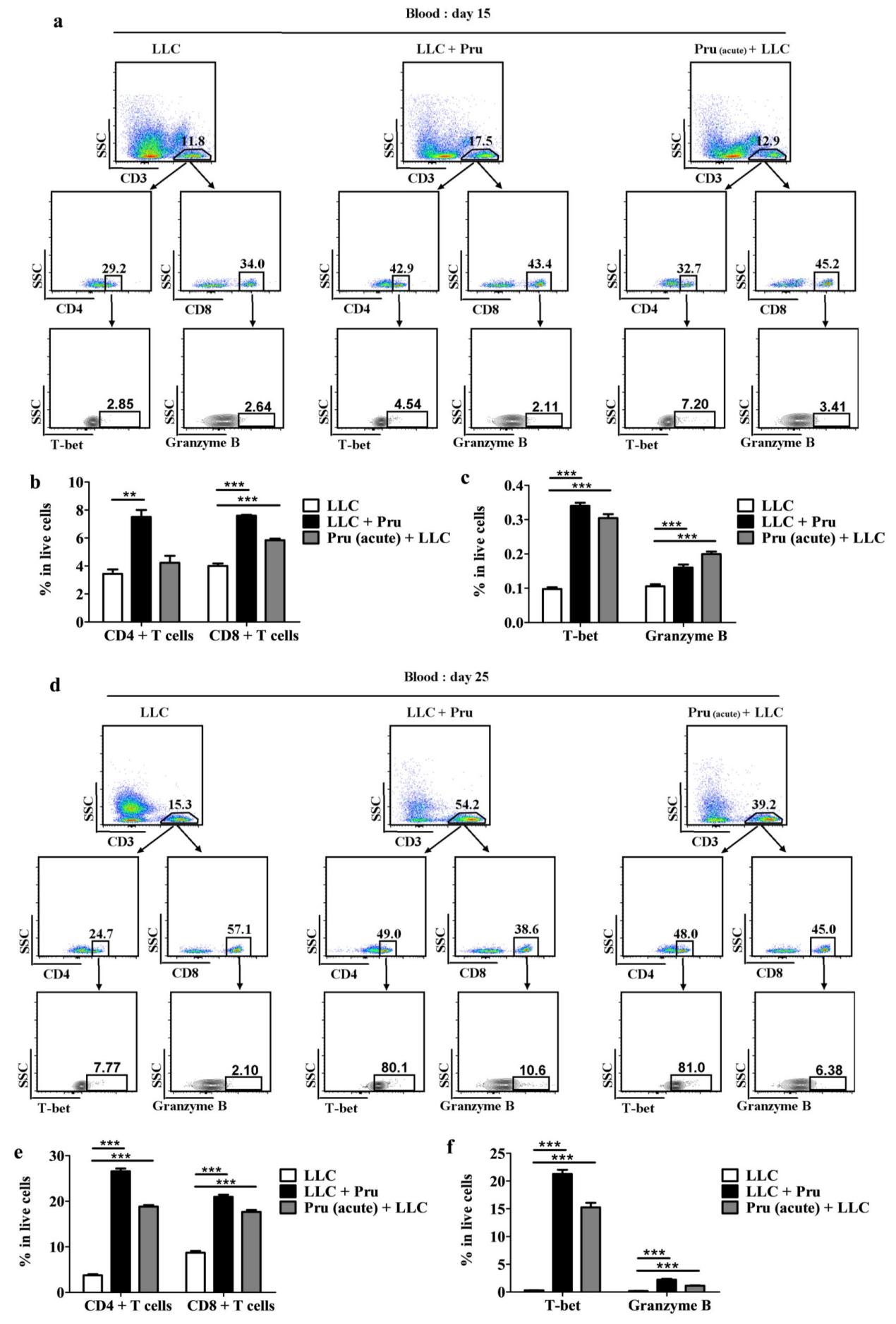
**

**Fig. S2.** The effect of *T. gondii* acute infection on T cells in peripheral blood (model III). (a) On the 15^th^ day after the mice injected LLC cells, CD3^+^ T cells in peripheral blood accounted for the proportion of total cells; CD4^+^ T cells as a percentage of CD3^+^ T cells; CD8^+^ T cells as a percentage of CD3-T cells; Th1 CD4^+^ T cells as a percentage of CD4^+^ T cells; Granzyme B as a percentage of CD8^+^ T cells. (b) On the 15^th^ day after the mice were injected with LLC cells, CD4^+^ T cells in peripheral blood accounted for the proportion of total cells; CD8^+^ T cells accounted for the proportion of total cells. (c) On the 15^th^ day after the mice were injected with LLC cells, Th1^+^ CD4^+^ T cells in peripheral blood accounted for the proportion of total cells; and Granzyme B accounted for the proportion of total cells. (d) On the 25^th^ day after the mice injected LLC cells, CD3^+^ T cells in peripheral blood accounted for the proportion of total cells; CD4^+^ T cells as a percentage of CD3^+^ T cells; CD8^+^ T cells as a percentage of CD3-T cells; Th1 CD4^+^ T cells as a percentage of CD4^+^ T cells; Granzyme B as a percentage of CD8^+^ T cells. (e) On the 25^th^ day after the mice were injected with LLC cells, CD4^+^ T cells in peripheral blood accounted for the proportion of total cells; CD8^+^ T cells accounted for the proportion of total cells. (f) On the 25^th^ day after the mice were injected with LLC cells, Th1 CD4^+^ T cells accounted for the proportion of total cells; and Granzyme B accounted for the proportion of total cells. Data are means ± SD (^**^ *P* < 0.01, ^***^ *P* < 0.001).

**
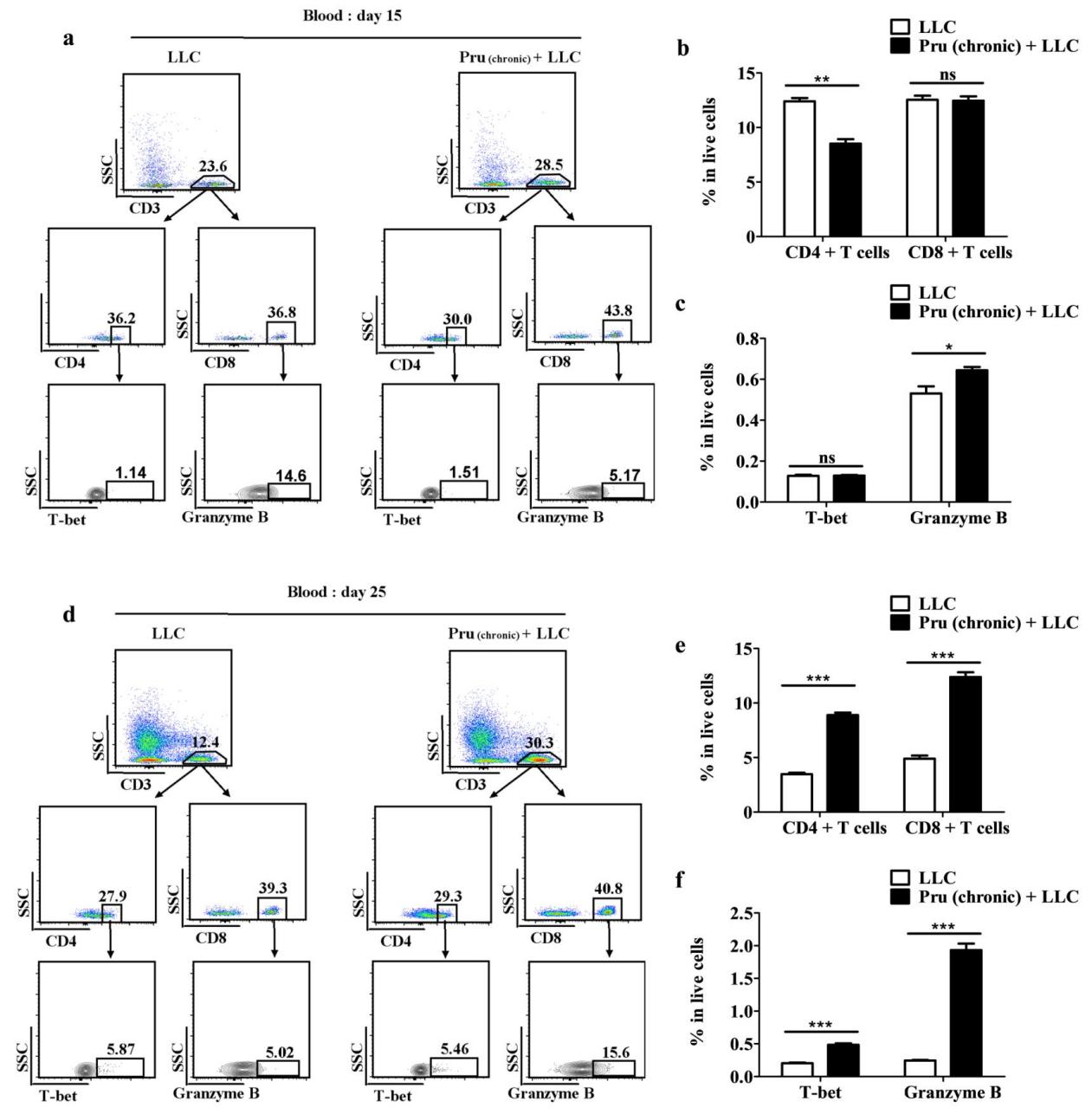
**

**Fig. S3.** The percentages of on effector T cells in peripheral blood (model III). (a) On the 15^th^ day after the mice injected LLC cells, CD3^+^ T cells in peripheral blood accounted for the proportion of total cells; CD4^+^ T cells as a percentage of CD3^+^ T cells; CD8^+^ T cells as a percentage of CD3-T cells; Th1 CD4^+^ T cells as a percentage of CD4^+^ T cells; Granzyme B as a percentage of CD8^+^ T cells. (b) On the 15^th^ day after the mice were injected with LLC cells, CD4^+^ T cells in peripheral blood accounted for the proportion of total cells; CD8^+^ T cells accounted for the proportion of total cells. (c) On the 15^th^ day after the mice were injected with LLC cells, Th1 CD4^+^ T cells in peripheral blood accounted for the proportion of total cells; and Granzyme B accounted for the proportion of total cells. (d) On the 25^th^ day after the mice injected LLC cells, CD3^+^ T cells in peripheral blood accounted for the proportion of total cells; CD4^+^ T cells as a percentage of CD3^+^ T cells; CD8^+^ T cells as a percentage of CD3-T cells; Th1 CD4^+^ T cells as a percentage of CD4^+^ T cells; Granzyme B as a percentage of CD8^+^ T cells. (e) On the 25^th^ day after the mice were injected with LLC cells, CD4^+^ T cells in peripheral blood accounted for the proportion of total cells; CD8^+^ T cells accounted for the proportion of total cells. (f) On the 25^th^ day after the mice were injected with LLC cells, Th1 CD4^+^ T cells accounted for the proportion of total cells; and Granzyme B accounted for the proportion of total cells. Data are means ± SD (^*^ *P* < 0.05, ^**^ *P* < 0.01, ^***^ *P* < 0.001).

**
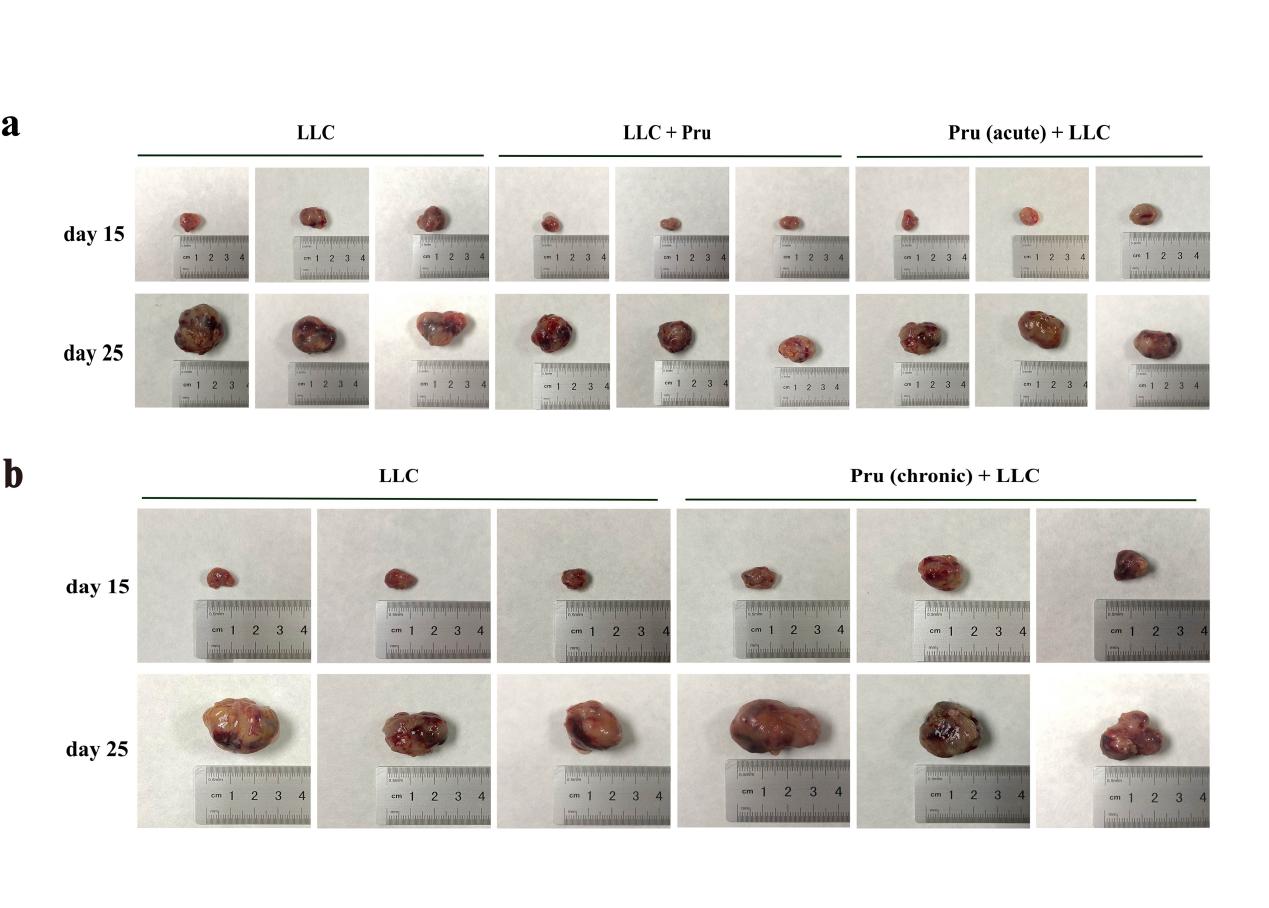
**

**Fig. S4.** Tumor images were captured from three C57BL/6J mice 15- and 25-days post injection of LLC-Luc cells.（a）Acute infection of *T. gondii* inhibits tumor growth (model II).（b）Chronic infection of *T. gondii* promotes tumor growth (model II).


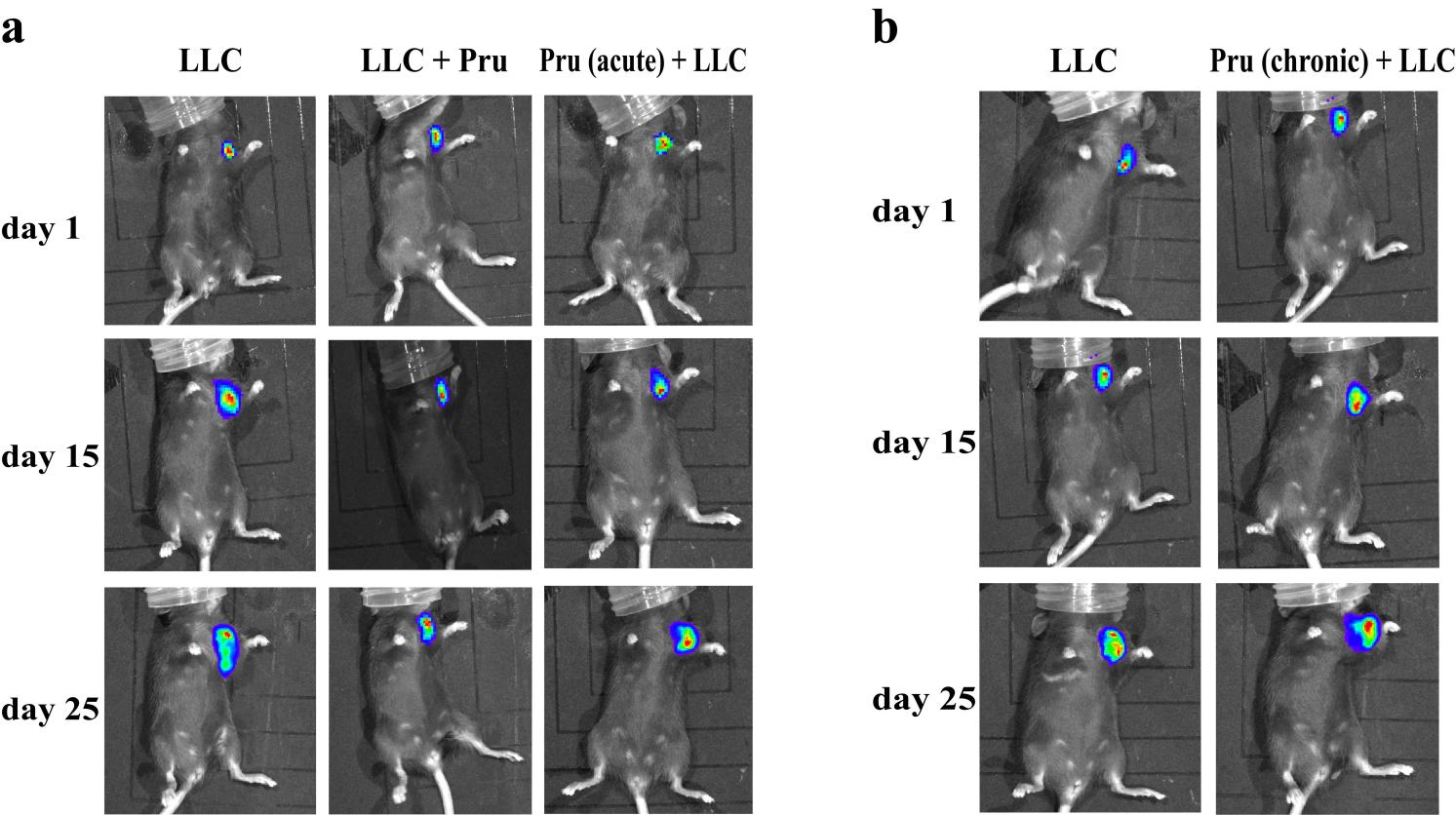


**Fig. S5.** Luminescence intensity pictures from bioluminescence imaging of live mice injected with LLC-Luc cells. (a) Bioluminescent imaging of tumor progression in LLC, LLC + Pru, and Pru (acute) + LLC groups. (b) Bioluminescent imaging of tumor progression in LLC and Pru (chronic) + LLC groups.

**Table S1. Quantitative real-time PCR specific primers set (model II)**

| Gene | Primer | 5’-3’ |
| --- | --- | --- |
| Sag1 | Sag1-F | 5’-GCTGTAACATTGAGCTCCTTGATTCCTG-3’ |
|  | Sag1-R | 5’-CCGGAACAGTACTGATTGTTGTCTTGAG-3’ |
| Bag1 | Bag1-F | 5’-AGTCGACAACGGAGCCATCGTTATC-3’ |
|  | Bag1-R | 5’-ACCTTGATCGTGACACGTAGAACGC-3’ |
| β-actin | β-actin-F | 5’-AGAGAAGCTGTGCTATGTTGCT-3’ |
|  | β-actin-R | 5’-GGAACCGCTCGTTGCCAATA-3’ |

| Tumor  Injection  Time | Chronic  *T. gondii*  Infection | KM Tumor Formation | | | BALB/c Tumor Formation | | |
| --- | --- | --- | --- | --- | --- | --- | --- |
|  |  | NO. Neoplasia | NO. Regression | Tumor Formation Rates %  (95 % CI) | NO. Neoplasia | NO. Regression | Tumor Formation Rates %  (95 % CI) |
| Week 1 | Positive | 16 | 0 | 100  (80.64, 100) | 16 | 0 | 100  (80.64, 100) |
|  | Negative | 16 | 0 | 100  (80.64, 100) | 15 | 1 | 100  (80.64, 100) |
| Week 2 | Positive | 13 | 3 | 81.25  (56.99, 93.41) | 16 | 0 | 100  (80.64, 100) |
|  | Negative | 15 | 1 | 93.75  (71.67, 98.89) | 15 | 1 | 93.75  (71.67, 98.89) |
| Week 3 | Positive | 9 | 7 | 56.25  (33.18, 76.9) | 11 | 5 | 68.75  (44.4, 85.84) |
|  | Negative | 8 | 8 | 50.00  (28, 72) | 14 | 2 | 87.5  (63.98, 96.5) |
| Week 4 | Positive | 7 | 9 | 43.75  (23.1, 66.82) | 11 | 5 | 68.75  (44.4, 85.84) |
|  | Negative | 5 | 11 | 31.25  (14.16 , 55.6) | 9 | 7 | 56.25  (33.18, 76.9) |

**Table S2. Four-week tumor formation rates in KM mice and BALB/c mice (model III)**
